# The Role of Bainite in Wear and Friction Behavior of Austempered Ductile Iron

**DOI:** 10.3390/ma12050767

**Published:** 2019-03-06

**Authors:** Fulin Wen, Jianhua Zhao, Dengzhi Zheng, Ke He, Wei Ye, Shen Qu, Jingjing Shangguan

**Affiliations:** 1 State Key Laboratory of Mechanical Transmission, College of Materials Science and Engineering, Chongqing University, Chongqing 400044, China; wfl_cqu@163.com (F.W.); happyzdzdsb@163.com (D.Z.); hk7313@hotmail.com (K.H.); 20160902042@cqu.edu.cn (W.Y.); 20160902005t@cqu.edu.cn (S.Q.); happysgjzll@163.com (J.S.); 2 National Engineering Research Center for Magnesium Alloys, Chongqing University, Chongqing 400044, China

**Keywords:** austempered ductile iron, friction and wear behavior, retained austenite

## Abstract

The austempered ductile iron was austenitized at 900 °C for 1 h and quenched in an isothermal quenching furnace at 380 °C and 280 °C, respectively. This paper aims to investigate the effects of bainite on wear resistance of austempered ductile iron (ADI) at different loads conditions. The micro-structure and phase composition of ADI was characterized and analyzed by metallographic microscope (OM), X-ray diffractometer (XRD) and scanning electron microscope (SEM) with energy dispersive spectroscopy (EDS). The results showed that the volume fraction of retained austenite in ADI is reduced with the increase of austenitizing temperature. Meanwhile, the two kinds of ADI samples showed varied wear resistance when they were worn at different loads conditions. For wearing at a load of 25 N, the wear resistance of ADI mainly depends on matrix micro-hardness. Thus, ADI with lower bainite structure has higher hardness and leads to better wear resistance. When wearing at a load of 100 N, the increase of micro-hardness of upper bainite was significant. As a consequence, upper bainite showed superior friction and wear behavior. It was also found that the form of wear behavior of ADI changed from abrasive wear to fatigue delamination as the wear load increased from 25 N to 100 N according to the observation on worn surface.

## 1. Introduction

Austempered ductile cast iron (ADI) has become an important engineering material because it provides an excellent combination of properties and low cost. Generally, the superior mechanical properties of ADI are mainly due to the mixed matrix of bainitic ferrite and carbon-enriched retained austenite, which was obtained by the heat treatment of austenitizing and isothermal quenching. The special microstructure provides attractive physical and mechanical properties, such as high tensile strength, ductility, fatigue strength, fracture toughness, and especially, wear resistance [1,2,3,4]. Because of these advantages, ADI has attracted significant attention in recent years due to its wide application in the automotive industry, defense and earth moving machineries, etc. [5,6,7,8].

In recent years, several studies have been reported to research the wear resistance of austempered ductile cast iron. Sahin et al. [9] found that the wear resistance of ADI samples is significantly higher than that of ductile iron samples because of its special microstructure. Sidjanin et al. [10] reported the mechanical properties of ADI and concluded that the optimal wear properties could be obtained if the microstructure consists of retained austenite and ausferrite. However, the researchers have not considered the effects of wear load conditions on the microstructural evolution of ADI. Zhang et al. [2] reported the effect of stress on the plastic deformation of retained austenite and found that initiation and propagation of cracks could be prevented by malleable austenitic membranes. Yang et al. [11] investigated the effects of the volume fraction of residual austenite on properties of ADI and excogitated the isothermal quenching process to control the volume frication of residual austenite. In addition, more and more researches indicate that the wear resistance of the material is closely related to the variation of worn subsurface [12,13,14,15,16]. For example, Li et al. [17] investigated the wear resistance of Inconel and found that Inconel 690 with a smaller grain size have much better wear resistance than that with larger ones. Amanov et al. [18] discussed the effects of surface modification on the wear resistance of Alloy 718 and concluded that the improvement of wear resistance could be ascribed to the increase of surface hardness. Olofsson et al. [19] investigated the wear resistance of quenching and tempering (QT) steel and ADI with an initial hardness of 635 Vickers microhardness (HV) and 315 HV. After a period of wear test, the relative wear-resistant performance of them was proved to be comparable.

The key objective of the present study is to reveal the response of two different kinds of bainite structure in the process of friction and wear experiment. Thus, the dry rolling—sliding wear tests of ADIs with variational load conditions were conducted. The relationship between the micro-hardness, dislocation density, stress-assisted phase transformation of retained austenite into martensite and wear resistance of ADI was investigated. Besides, the microstructure, wear morphology as well as the form of wear behavior of two typical types of ADIs are discussed in detail. It is expected to provide some theoretical support for the friction and wear mechanism of ADI in different wear conditions.

## 2. Experimental

### 2.1. Materials

Experimental samples were melted in a medium frequency induction furnace (Shuangping, ShenZhen, China) and cast in the form of 55 × 40 × 140 mm^3^. The chemical composition of as-cast ductile iron used was given in Table 1. The cast samples were divided into two groups, and both of them were austenitized at 930 °C for 1 h in a muffle furnace (Hongda, LuoYang, China). After austenitizing, the specimens were transferred to the furnace for isothermal quenching. They were rapidly quenched in 55%–45%NaNO_2_–KNO_3_ salt bath at 380 °C and 280 °C for 2 h, respectively. These two types of samples were recorded as ADI1 and ADI2, respectively.

### 2.2. Friction and Wear Test

**Figure 1:**
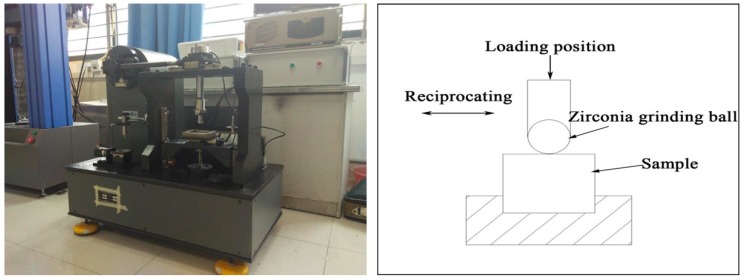
Frication and wear experiment device.

The ball disc reciprocating friction and wear tester (RTEC MFT5000, Rtec, Nanjing, China) was used to carry out the friction and wear tests by high speed reciprocating friction tests. Figure 1 show the installation drawings and the wear test specimens and schematic representation of the wear apparatus, respectively. The upper grinding ball was made of zirconia with a diameter of 5 mm, while the lower test block was made of two types of ADIs in the form of 30 mm × 20 mm × 10 mm. Before the wear tests, the samples were ground and accomplished with 800-grit silicon carbide paper and cleaned with acetone. During the wear tests, the test block stayed in the same position, while the grinding ball kept reciprocating motivation. To investigate the effect of different load levels on the wear-resisting performance of ADI, the loads applied on the samples were 25, 65, 85, and 100 N at a total sliding time of 3600 s, and a sliding frequency of 5 Hz. Moreover, all the test samples were ensured to be conducted under dry conditions in a normal laboratory atmosphere (55%–65% relative humidity, 20–26 °C). Meanwhile, a data collection system was used to gather the signals and information from wear tests. The averaged steady-state friction coefficient of the test samples was calculated by utilizing the results of at least three specimens of each load condition.

### 2.3. Characterization

After the heat treatment, the specimens were sectioned and polished, then etched by the 4% nital. The microstructure was observed by a metallographic microscope (OM, AxioCamMRc5, Oberkochen, Germany, Carl Zeiss). The study of the morphology of the worn surface of two kinds of ADI after friction and wear test was conducted under the TESCANVEGA III scanning electron microscope (SEM, TESCAN, Brno, Czech Republic). The distribution of chemical compositions of abrasive debris was measured using an energy dispersive spectroscopy (EDS, Ox 4796, Oxford Instrument, High Wycombe, UK) detector.

Phase quantitative analysis was performed using a D/max2500PC X-ray diffractometer XRD (PANalytical B.V., Almelo, the Netherlands, employing Cu-Kα radiation, λ = 1.544 Å) before and after wear tests. The tube acceleration voltage was 40 kV, the current was 40 mA, and 2θ values were between 0 to 90^°^. Measuring the {111}, {200}, {220} diffraction peaks of austenite and {200}, {211} diffraction peaks of ferrite/martensite to calculate the residual volume fraction of the austenite by the equation as reported in [20]:

(1) $$Vi=\frac{1}{1+G\left(I\alpha /I\gamma \right)}$$

where *I_α_* and *I_γ_* are the integral strength of diffraction peak of ferrite/martensite and austenite respectively. *V_i_* is the volume fraction corresponding to each diffraction peak of austenite. The value of G is selected as follows: 2.5 for *I_α(200)_/I_γ(200)_*, 1.38 for *I_α(200)_/I_γ(220)_*, 2.02 for *I_α(200)_/I_γ(111)_*, 1.19 for *I_α(211)_/I_γ(200)_*, 0.06 for *I_α(211)_/I_γ(220)_*, 0.96 for *I_α(211)_/I_γ(111)_*.

The carbon content of austenite was determined by the equation as reported in [21]:

a_0_ = 3.555 + 0.44x (2)

where a_0_ is lattice parameter of austenite which was calculated based on the {111}, {200} diffraction peaks of austenite and x is carbon content of austenite in [wt.%].

HX-1000TM/LCD Vickers micro-hardness tester (HTZ, Shanghai, China) was used to conduct the Vickers microhardness (HV) tests on the surface of ADI samples under a load of 2.94 N and dwell time of 10 s. The average hardness values of the ADI samples were calculated in accordance with at least three readings. An adequate space distance indent should be guaranteed to avert the mutual interference.

## 3. Results and Discussion

### 3.1. The Microstructure and Wear Rate of ADIs 

**Figure 2:**
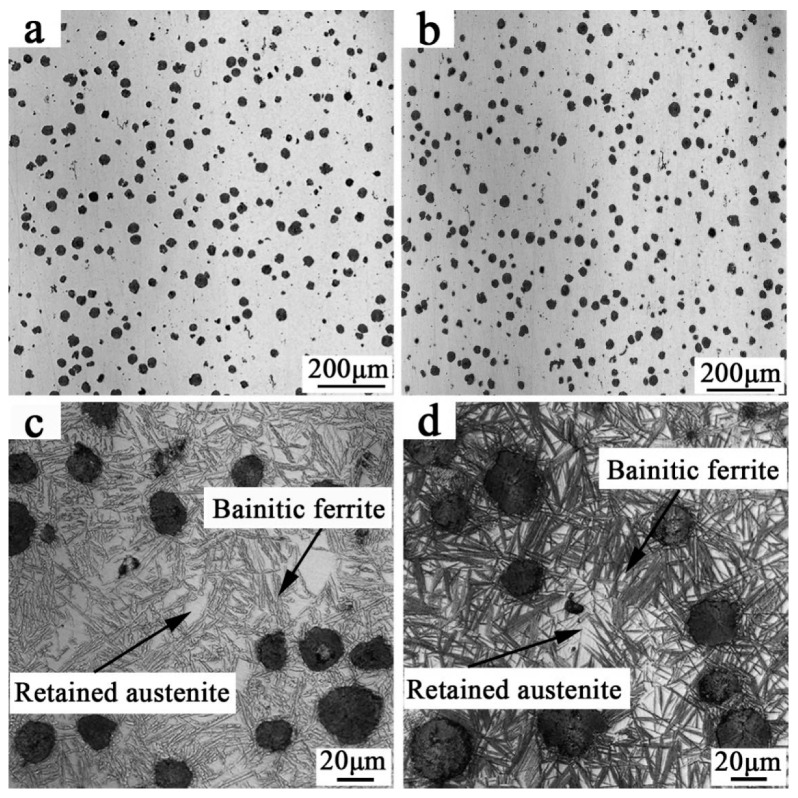
The microstructure of austempered ductile iron (ADIs) observed by an optical microscope (**a**,**b**) as-cast; (**c**) ADI1; (**d**) ADI2.

Figure 2a,b show the microstructure of as-cast sample of ADI1 and ADI2. The software of Image-Pro Plus (Version 6.0) was used to characterize the feature of the graphite nodule. As shown, the graphite nodule and matrix structure can be distinguished by their different tinted grayscale and, thus, they can be colored by different colors using this software. Then the volume fraction of graphite nodules could be confirmed by counting its area percentage in the figure, and the calculated values are 0.86% and 0.91%, respectively.

Figure 2c,d show the typical optical micro-graphs of ADI1 and ADI2 which was austempered at 380 °C and 280 °C, respectively. It can be observed that the morphology of bainite structure evolved from more plate-like (feathery) to needle-like (acicular) when the isothermal quenching temperature was reduced from 380 °C to 280 °C [14,15]. The main reason for the different morphology of the bainite structure was the different diffusion rate of carbon. At 380 °C, the speed of diffusion of carbon dissolved from ferrite into the surrounding austenite was faster than the speed of the nucleation and growth of ferrite. Thus, the microstructures consist of austenite + upper bainite ferrite (ausferrite) which can be obtained by inhibiting the formation of carbides. However, as the temperature decreased to 280 °C, the speed of diffusion of carbon dissolved from ferrite into the surrounding austenite was slower than the speed of the nucleation and growth of ferrite. Thus, the microstructures consisted of carbides and precipitates.

The wear performances of the ADI1 and ADI2 at different loads conditions were evaluated using the ball disc reciprocating friction and wear tester.

**Figure 3:**
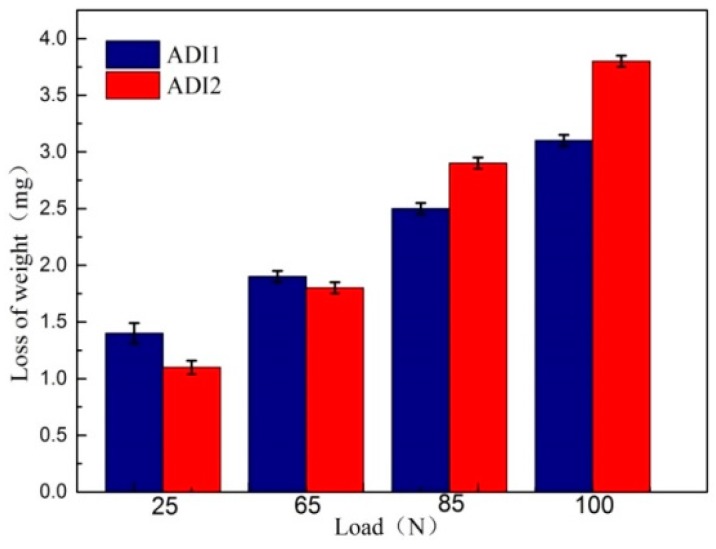
Wear rate of ADIs under different load conditions.

Figure 3 demonstrates the accumulated wear rates of ADIs after 18,000 revolutions under different wear loadings. As shown, the variation of wear rate is closely related to the wear load. When the wear load is 25 N and 65 N, ADI1 reveals a higher wear rate which means lower wear resistance. However, when the wear load exceeds 65 N, ADI2 reveals the higher wear rate which means lower resistance. It indicates that the relative value of wear resistance changes with the increase of wearing load. Additionally, wear resistance may also relate to the volume fraction of retained austenite which indicates that the wear resistance depends on the amount of retained austenite [22].

### 3.2. The Microstructure of ADI after Wearing Tests

**Figure 4:**
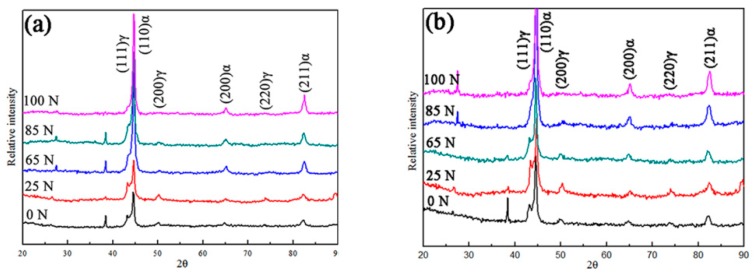
X-ray diffraction diagrams for ADIs in the process of wear tests (**a**) ADI1; (**b**) ADI2.

XRD was used to accurately calculate the volume fraction of austenite before and after wear tests. The X-ray diffraction patterns of the ADI1 and ADI2 after the wear tests at various load conditions are shown in Figure 4. The variations of relative intensity of the austenite and ferrite/martensite peaks reflect the evolutions of transformed phases. As shown in Figure 4, the diffraction peaks {111}, {200}, and {220} of austenite in ADI1 and ADI2 at the load of 100 N are lower and relatively thinner than that at 25 N. Meanwhile, the ferrite/martensite peaks {200} and {211} of ADI1 and ADI2 at the load of 100 N were gradually widened than that at 25 N. The volume fractions of the austenite were determined by Formulas (1) and (2) and XRD results mentioned above. Table 2 demonstrates the change of volume fraction of retained austenite and its carbon content before and after wear test. As shown, the volume fraction of retained austenite in ADI1 and ADI2 was about 26.2% and 13.5%, respectively, before wearing tests, respectively. It indicates that the volume fraction of retained austenite increased with the increase of austempering temperature. After wearing at 100 N, the final volume fraction of retained austenite in the surface microstructure of ADI1 and ADI2 was 18.4% and 8.3%, respectively. It demonstrates that the variation of the volume fraction of retained austenite of ADI1 was more obvious than that of ADI2. Based on the variation of the ferrite/martensite peaks {200} and {211} of ADI1 and ADI2, it can be found that stress-assisted phase transformation of retained austenite into martensite phenomenon is more obvious in ADI2. Meanwhile, the carbon content of retained austenite before and after wearing tests is shown in Table 2. Before wearing tests, the carbon content of retained austenite of AD1 and ADI2 was 1.51% and 1.82%, respectively. Thus, the retained austenite in ADI1 showed better stability than ADI2. Therefore, the retained austenite in ADI1 is more likely to occur stress-assisted phase transformation of retained austenite into martensite.

**Figure 5:**
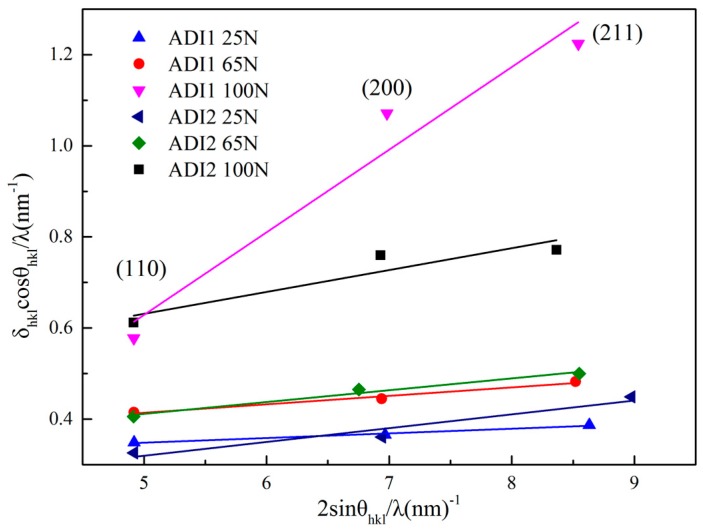
Relationship between δ_hkl_cosθ_hkl_/λ and 2sinθ_hkl_/λ.

In general, severe plastic deformation of the subsurface structure during the friction process led to the increase of dislocation density in the worn surface of ADI1 and ADI2. The method proposed by Williamson and Hall (WH) using X-ray diffraction patterns was widely used to measure the dislocation density of metal microstructure [23,24,25]. In this study, the diffraction peak broadening model caused by the variation of grain size and strain was adopted to calculate the dislocation density, and it could be described by effective micro-strain parameter e in [23,26]:

(3) $$\delta_{\mathrm{hkl}}\frac{cos\theta_{hkl}}{\lambda }\approx 2e\frac{sin\theta_{hkl}}{\lambda }$$

where $\theta_{hkl}$ represents the position of the diffraction peak; λ represents wavelengths; $\delta_{\mathrm{hkl}}$ represents the width of the diffraction peak, and the value of it can be obtained by the following formula:

(4) $$\delta_{\mathrm{hkl}}=\sqrt{\delta_{\mathrm{hkl}\;m}{}^{2}-\delta_{\mathrm{hkl}\;0}{}^{2}}$$

where $\delta_{\mathrm{hkl}\;m}$ represents the full width at half maximum of diffraction peaks of ADI samples after wearing; $\delta_{\mathrm{hkl}\;0}$ represents the full width at half maximum of diffraction peaks of ADI samples before wearing. Diffraction peaks of the ferrite {110}, {200}, and {211} were selected to calculate the values of $\delta_{\mathrm{hkl}}cos\theta_{hkl}$/λ and $2sin\theta_{hkl}$/λ. After that, the values of effective micro-strain parameter e could be obtained by linear fitting the slope of linear function and are shown in Figure 5.

According to Figure 5, the slope of each fitting curve represented the values of effective micro-strain parameter e of ADI samples during tribological testing. With the wear load increased from 25 N to 65 N and 100 N, the values of effective micro-strain parameter were added gradually. What is more, the change of the values of effective micro-strain parameter e was more obvious in ADI1 than that of ADI2 when the wear load was 100 N.

According to Williamson’s study, assuming that the variation of dislocation density is caused by lattice distortion only, the relationship between the dislocation density and the micro-strain exists as follows [23]:

(5) $$\rho \;=\;14.4\frac{e^{2}}{b^{2}}$$

where *b* is the Burgers vector; *e* represents effective micro-strain shown in Figure 4.

**Figure 6:**
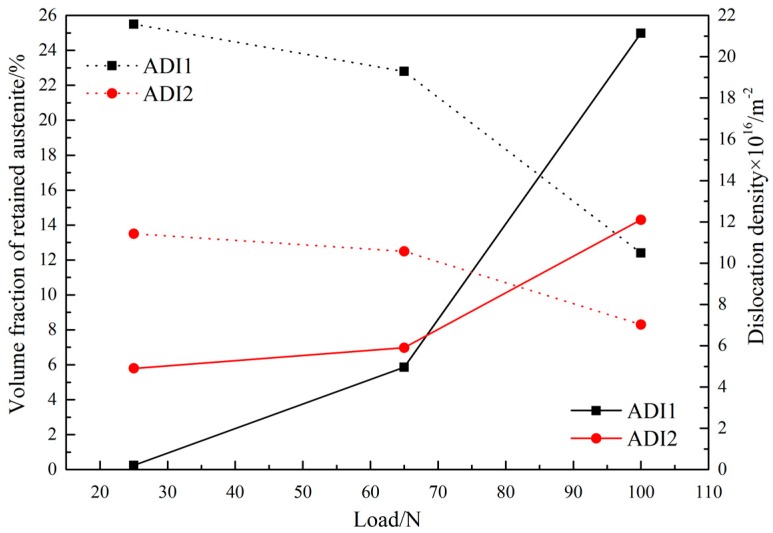
The volume fraction of retained austenite and dislocation density of ADI worn surface under different load conditions.

Figure 6 shows the variation of the volume fraction of retained austenite and dislocation density of ADIs at different wear load conditions. As shown, when the wear load increased from 25 N and 65 N, the variation of the volume fraction of retained austenite in ADI1 and ADI2 is indistinctive. However, when the loads of wear tests increased to 100 N, the volume fraction of retained austenite in ADI1 reduced significantly. Meanwhile, as the wear load increased from 25 N and 65 N, the dislocation density changed from 2.02 × $10^{15}\;m^{-2}$ to 5.1 × $10^{16}\;m^{-2}$ for ADI1 and from 5.3 × $10^{16}\;m^{-2}$ to 6.8 × $10^{16}\;m^{-2}$ for ADI2. It indicates that the dislocation density in the microstructure of ADIs increased with the decrease of austempering temperature. However, when the wear load increased to 100N, the dislocation density of ADI1 and ADI2 increased from 5.3 × $10^{16}\;m^{-2}$ to 2.1 × $10^{17}\;m^{-2}$ and from 6.8 × $10^{16}\;m^{-2}$ to 1.2 × $10^{17}\;m^{-2}$, respectively, under the wear load of 100 N. It means that the dislocation density in worn subsurface of ADI1 exceeded that of ADI2. Therefore, as the wear load increased from 25 N to 100 N, a higher density of dislocation generated in ADI1 because of a larger micro-strain parameter e. As a result, the fracture strength of the worn subsurface structure of ADI1 is higher than that of ADI2 which leads to a lower wear rate of ADI1 at a wear load of 100 N. Furthermore, according to previous studies by others, the dislocation was proved to become the potential nucleation position of martensite phase transformation [27]. To some extent, the increased dislocation density has benefits for the stress-assisted phase transformation of retained austenite into martensite.

**Figure 7:**
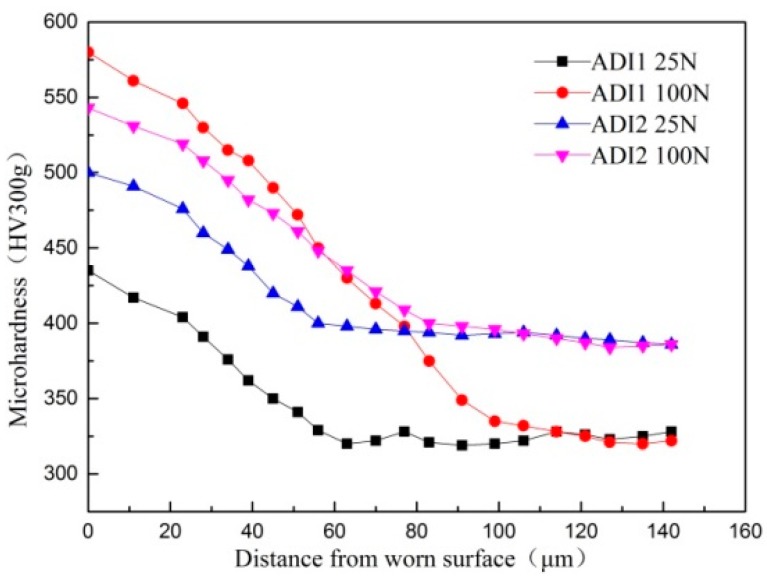
Variation of micro-hardness along the depth from the worn surface for ADIs.

Figure 7 shows the micro-hardness of ADIs in the subsurface region measured on the cross-section before and after wearing tests at different loads conditions. It can be generally found that micro-hardness of ADI1 is smaller than that of ADI2 before wearing tests and that is in accordance with previous analysis in which ADI1 has lower hardness because it contains more volume fraction of softer phase retained austenite than ADI2. After wearing tests at the wear loads of 25 N and 100 N, the microhardness of ADI1 near the worn surface increases from 347 HV to 424 HV and 347 HV to 547 HV, respectively, and the microhardness of ADI2 near the worn surface increases from 406 HV to 501 HV and 406 HV to 573 HV, respectively. It is obvious that the increase of micro-hardness in ADI1 was more remarkable when the wear load was 100 N. According to previous reports, the increase of hardness can be explained by two major reasons. On the one hand, the subsurface deformation was caused by the wear loads of 100 N applied on the friction surface. Strain hardening of the matrix at the subsurface region leads to an increase of hardness. On the other hand, as the retained austenite transformed into martensite, it contributed to the increase of hardness at the subsurface region.

### 3.3. Wear Behaviour of ADI

**Figure 8:**
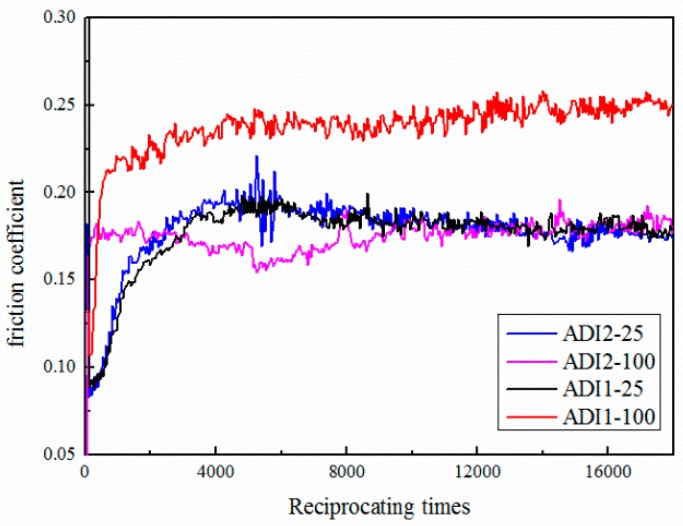
The friction coefficient of ADIs under different load condition.

Figure 8 shows the steady-state coefficient of friction at a constant frequency (5 HZ) and reciprocating times (18,000) for ADIs at the wear load of 25 N and 100 N. As shown, under the conditions of dry and continuous cooling, the average values of coefficient of friction were different from each other. Wearing at 25 N, ADI1 and ADI2 revealed stable and commensurate coefficient of about 0.17. The samples of ADI1 worn at 100 N showed the highest friction coefficient level of about 0.23. However, the friction behavior of ADI2 revealed lower friction coefficient values of about 0.16. This variation in the friction coefficient can be attributed to the variation in hardness. Some researches indicated that the values of the steady-state friction coefficient increased with the increase of a sample’s hardness [28]. Meanwhile, the role of graphite in the matrix of ADI should not be ignored. In general, graphite in the ADI matrix acted as a lubricant when ADIs wore against the grinding ball. Therefore, when the wear loading was 100 N, ADI2 with a lower bulk hardness might undergo more severe plastic deformation resulting in a larger amount of graphite smeared on the contact surface, which resulted in the lowest coefficient of friction.

### 3.4. Wear Forms at Different Wear Loads 

**Figure 9:**
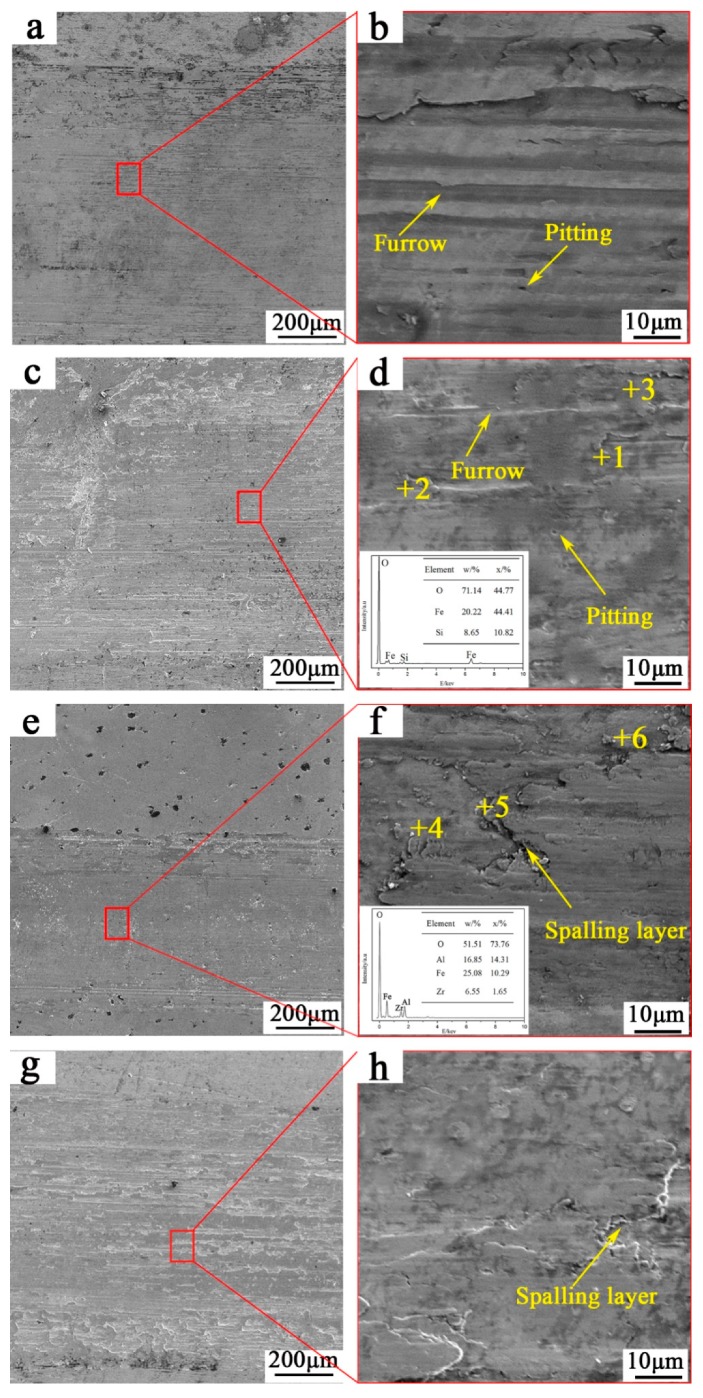
Observation and energy dispersive spectroscopy (EDS) on worn surface (**a**) ADI1 25 N; (**b**) ADI2 25 N; (**c**) ADI1 100 N; (**d**) ADI2 100 N. (**e**,**f**) ADI1 100 N; (**g**,**h**) ADI2 100 N.

Figure 9a–d show the SEM micrographs of worn surfaces of ADI1 and ADI2 at wear loads of 25 N. As shown, furrow morphology with cutting traces can be seen on the worn surface which means groups of ADIs undergo wear by plastic deformation. However, the depth of the furrow on ADI1 is deeper than that of ADI2. It may be attributed to a larger volume fraction of retained austenite and a smaller micro-hardness in ADI1. In contrast with ADI1, furrows on the worn surface of ADI2 are discontinuous and always terminate at some point. Figure 9d (Point 1–3) shows the average values of composition analysis of granular matter under a load of 25 N. The results of EDS showed it is iron oxides. It is considered that the wear mechanism of ADIs is mainly abrasive wear, accompanied by a small amount of oxidation wear.

Figure 9e–h shows the SEM micrographs of the worn surface of ADIs at wear loads of 100 N. As shown, scaly peeling layer can be seen on the worn surface because of the increase of shear stress in the worn subsurface. Meanwhile, the surface of ADI1 and ADI2 was rolled repeatedly by grinding balls so that the crack initiated easily. Once the crack is formed, its rapid propagation will lead to a fatigue stratified fracture of the surface microstructure. As Figure 6 shows, after wearing at loads of 100 N, both the dislocation density and micro-hardness of ADI1 exceeded that of ADI2. Thus, the fracture strength of worn surface microstructure of ADI1 was higher than that of ADI2. Figure 9f (Points 4–6) shows the average values of composition analysis of wear debris under a load of 100 N of ADI. The results of EDS showed wear debris contain Al, Zr, O, and Fe. It showed that the material in the grinding pit is transferred to the ball and wear mechanism of ADIs is mainly a fatigue delamination fracture.

## 4. Conclusions

The effects of microstructure on wear resistance and mechanical properties of ADI were investigated in this study. The main conclusions acquired are listed as follow:

1. When the austempering temperature increases from 280 °C to 380 °C, the microstructure of ADI changes from a needle-like (acicular) to a more plate-like (feathery), gradually.
2. The volume fraction of retained austenite increases with the increase of austempering temperature. Meanwhile, after wear testing, more volume fraction of retained austenite in ADI1 was observed which occurred because of the stress-assisted phase transformation of retained austenite into martensite phenomenon.
3. The dislocation density of ADI2 is larger than that of ADI1 when the wear loading is 25 N and 65 N. However, as the wear loading increased to 100 N, the dislocation density in the worn subsurface of ADI1 exceeds that of ADI2.
4. The occurrence of more martensite and the increase of dislocation density in the worn subsurface of ADI1 led to an obvious increase of its Vickers microhardness, which improved the wear resistance of itself.
5. The Vickers microhardness of ADI1 and ADI2 in the subsurface region increases with the increase of wear loading. According to the results of wear rate and coefficient of friction, the wear resistance of ADI2 is superior to that of ADI1 when the wear loading is less than 65 N. However, as the wear loading increased to 100N, the wear resistance of ADI1 exceeds that of ADI2.
6. According to the SEM micrographs of the worn surface and EDS of worn debris, the wear mechanism of ADIs is mainly abrasive wear when the wear loading is 25 N but fatigue delamination fracture when the wear loading is 100 N.

## Figures and Tables

**Table 1:** Chemical composition of the test materials.

Elements	C	Si	Mn	Mo	Ni	P	S	Re
Compositions (%)	3.61	2.49	0.25	0.30	1.70	≤0.05	≤0.015	≤0.02

**Table 2:** The volume fraction of retained austenite and its carbon content.

Samples	Before Wear Test		After Wear Test (25 N)		After Wear Test (100 N)	
	ADI1	ADI2	ADI1	ADI2	ADI1	ADI2
Volume fraction of retained austenite [%]	26.2 ± 1.0	13.5 ± 1.0	25.5 ± 1.0	9.5 ± 1.0	18.4 ± 1.0	8.3 ± 1.0
Carbon content in austenite X^r^ [%]	1.51 ± 0.05	1.82 ± 0.05	1.53 ± 0.05	1.87 ± 0.05	1.55 ± 0.05	1.86 ± 0.05
